# First case of highly pathogenic H5N1 avian influenza virus in Spain

**DOI:** 10.1186/1746-6148-4-50

**Published:** 2008-12-10

**Authors:** M Barral, V Alvarez, RA Juste, I Agirre, I Inchausti

**Affiliations:** 1 Department of Production and Animal Health, NEIKER-Tecnalia-Instituto Vasco de Investigación y Desarrollo Agrario, Berreaga 1, 48160 Derio, Bizkaia, Spain; 2 Diputación Foral de Gipuzkoa, Pza. de Gipuzkoa s/n, 20004 Donostia-San Sebastián, Gipuzkoa, Spain; 3 Diputación Foral de Bizkaia, Avda. Lehendakari Agirre, 9, 48014 Bilbao, Bizkaia, Spain

## Abstract

**Background:**

The H5N1 strain of avian influenza virus has been involved in severe mortality in domestic poultry, and has also been found in different species of wildlife in Europe. The Basque Country avian influenza surveillance program began sample collection and processing the fall of 2005.

**Results:**

Here we report the first confirmation of the presence of highly pathogenic H5N1 strain in a Great Crested Grebe (*Podiceps cristatus*) found dead in a pond near Vitoria in the Basque Country on the North of Spain. Regarding the survey for generic influenza type A virus, we have obtained positive results in about 8% of more that 3500 birds examined.

**Conclusion:**

We think that the self-limiting nature of our finding and others proves that certain regions have ecological, geographical and climatological features that make it difficult for the H5N1 virus to spread [1] and cause disease at least in the large scale scenario that has been worrying human and animal health authorities during the last years.

## Background

The H5N1 strain of avian influenza virus has been circulating in different countries since its first detection in China in 1996 causing severe damages to the fowl industry in some regions. In Europe, it was first detected during fall of 2005 in Croatia and Romania, according to the World Organisation for Animal Health (WOAH). During 2006, it was reported from an important number of countries including Germany, Denmark, France, Greece, Italy, Sweden and Czech Republic where it was involved in severe mortality in domestic poultry, and/or in different species of wildlife. In spite of the Iberian Peninsula being an important area in migratory routes and holding at given moments around 300.000 migratory birds, no case of H5N1 infection had been ever detected. Here we report the first confirmation of the presence of this viral strain in a Great Crested Grebe (*Podiceps cristatus*) found dead in a pond near Vitoria in the Basque Country on the North of Spain, as well as the results of generic influenza type A testing in the Basque Country.

## Results and discussion

A grebe reportedly found dead in Salburua pond and registered in the laboratory on 30^th ^June 2006, tested positive (Ct = 39) in a screening step with influenza A type matrix gene real time reverse transcription PCR (RRT-PCR) using the primers described by Spackman and others 2002 [2]. Then it was submitted to the H5 and H7 specific RRT-PCR [2] which came out positive at 29 ct and negative respectively. A H5N1 commercial conventional multiplex RT-PCR was run (Veredus Laboratories^©^) that yielded also a positive result for H5 and for N1.

At the same time five embryonated Specific Pathogen Free (SPF) eggs of 9–11 days of incubation were inoculated with a swab following the OIE recommendations [3,3]. At 24 h postinoculation four embryos out of five died and only the allantoic fluid of the surviving egg was positive to slide and plate erythrocyte hemagglutination (HA). After that, the positive allantoic fluid was tested for hemagglutination inhibition (HI) to determine the HA subtype [3]. The HI results of the allantoic fluid were negative for H7 and positive at 1/64 to 1/128 dilutions for H5. At 24–48 h postinoculation of a second and third passage of the surviving embryo, the allantoic fluids were collected and tested for HA and HI, and all of them were positive with H5 reference positive serum. Also, the five SPF eggs allantoic fluids of the first passage were submitted to conventional RT-PCR A type [4] and H5 subtype (Avian Influenza European Reference Laboratory, Weybridge) that yielded positive results in all cases.

The haemagglutinin and neuraminidase PCR products were sequenced [5] and the partial sequences deposited in GenBank (Accession numbers: EU636810 and EU636811). Its high pathogenicity was first determined by a conventional PCR based in the protocol described by Payungporn and others in 2006 [6], and after that by the study of the sequence of the cleavage site (GERRRKKR*GLF) that characterised this strain as of highly pathogenic.

**Figure 1:**
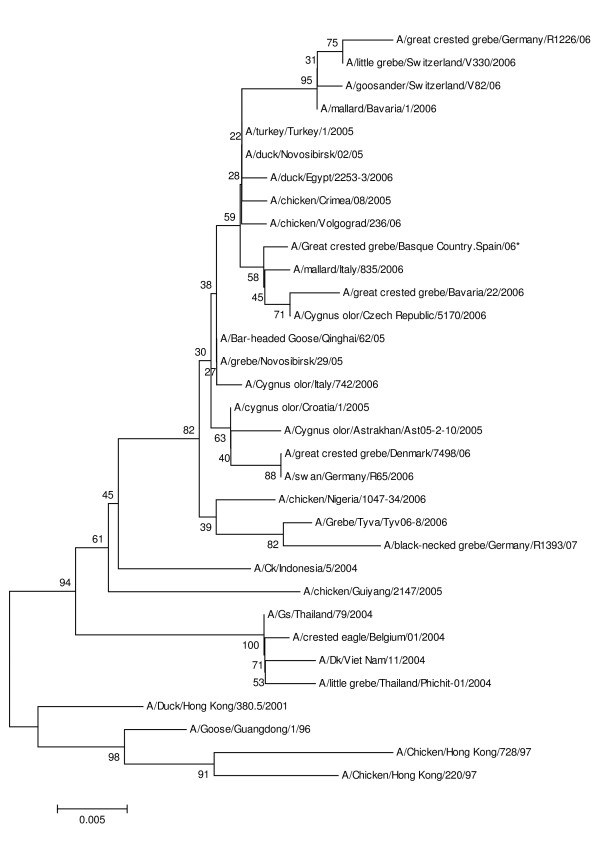
**Phylogenetic analysis of the HA nucleotide sequence of some H5N1 strains**. The A/great crested grebe/Basque Country.Spain/06* virus sequence was generated in this study. Phylogenetic analysis of the NA nucleotide sequence offered similar results with the A/great crested grebe/Basque Country.Spain/06* virus in a cluster that includes A/mallard/Italy/835/2006 and A/Cygnus olor/Czech Republic/5170/2006. The A/great crested grebe/Bavaria/22/2006 NA nucleotide sequence was not available.

The sequences obtained were compared with those already available in GenBank database by nucleotide sequence homology searches made at the network server of the National Center for Biotechnology Information (NCBI) using BLAST. Multiple sequence alignments were performed using the program AlignX (Vector NTI 8.0 suite, InforMax, North Bethesda, MD, USA) with an engine based on the ClustalW algorithm [7]. Finally sequences extending from nucleotide 1 to 562 of the HA gene (EU636811) and from nucleotide 60 to 845 of the NA gene (EU636810) were included in the analysis. Similarity matrices were then constructed from aligned sequence data by the Kimura's two-parameter model [8]. Phylogenetic trees were constructed by the neighbour joining method [9] as implemented in the Mega 4 package [10] (Figure 1). Stability or accuracy of inferred topologies were assessed via bootstrap analysis of 1000 replicates.

These phylogenetic analyses showed that the great crested grebe virus isolated in the Basque Country belongs to the Asian H5N1 HPAI virus lineage. The closest relatives are viruses found in Italy, Czech Republic and Bavaria in 2006.

A sample was also sent to the Spanish National Reference Laboratory (SNRL) for official confirmation.

Regarding the survey for generic influenza type A virus, we have obtained positive results in about 8% of all wild bird samples examined. None of them were positive in the H5 or H7 RRT-PCR excluding the Great Crested Grebe case. Currently we are sequencing these positive samples in order to determine influenza virus subtype (data not shown).

The results of our investigation as confirmed by the SNRL have been officially registered by OIE as the first cases of the H5N1 avian influenza subtype in the Iberian Peninsula. It is very striking that it has first been detected in the Basque Autonomous Community which according to Spanish Ministry of Agriculture, Fisheries and Food data holds only 3% of the migratory birds in Spain and has processed less than 4% (3,2–3,5%) of the samples examined in the whole country. Great Crested Grebe are regarded as sedentary in the Basque Autonomous Community with 47 breeding pairs and 240 wintering birds registered in 2006 census [11,12].

Our results on type A influenza virus prevalence in wild birds are in agreement with estimates from some authors [13]. However, they seem to disagree with others [14] in the sense that we have found around ten times more positives than those reported.

All these disagreements are difficult to explain and require a closer study. One possible explanation is that our extraction method has a higher sensitivity to detect low levels of virus as we use a larger fraction of the total amount of available sample than other protocols [14]. Other reasons that might contribute are poor sample preservation and transport imposed by the larger dispersion and distances of sampling locations with respect to the laboratory processing the samples, and less likely, absence of this virus in the larger wetlands in central and south Spain.

In relation as to how this Great Crested Grebe became infected the most likely hypothesis is contact with subclinical anatid carriers as wild ducks have demonstrated that they can excrete virus without showing clinical signs. [15]. On the other hand, it could be possible that the grebe became infected in another European country and after that arrived to Salburua pond to feather moult, where it died. Some water birds undergo a moult migration moving to sites where they are lower risk of predation and have food enough. Such movements generally occur between late June and August with timing variations attending species and region [16]. Another unlikely alternative is arrival through commercial duck imports since an unconfirmed H5 result was reported a few months before in a neighbouring region [17].

Anyway, it is worth to point that according to European Union annual reports , this case, which seems to be the most south-westerly case in Europe, could represent a change of host species and seasonality, since it was one of the first cases in a grebe that was followed in 2007 by 269 cases (87.3% of wild birds cases) all throughout Europe in summer. Up to then the most affected species were swans (62.9%) and the predominant season winter.

On the other hand the criteria of considering the generic matrix RRT-PCR as a screening on whose results decide whether or not to submit the samples to further more specific testing needs to be reviewed, as some laboratories only accept positive results under Ct 36 [14] and can also contribute to overlooking earlier cases of H5N1 infection in wild birds.

We think that surveillance needs to be increased and the methods reviewed. Lack of striking increases in mortality supports the hypothesis that the virus is widely spread among subclinical carriers probably of the anatidae family and that only sporadically it reaches more susceptible species where some mortality occurs. For wild birds, given its over-dispersion and lack of close contact, these cases are probably rare and self-limiting. If the virus happens to reach a domestic bird's farm, the spread will be massive and will cause high mortality. Lack of such episodes in Spain indicates that farms have enough biosecurity measures in place, although there were no commercial farms in the 3 Km protection area or the 10 Km surveillance area around where this case was detected. The biggest risk of human infection seems to be highly limited not to wild birds' infection, but to domestic large outbreaks and in situations of animal-human very close contact and probably also high humidity and other host predisposing factors.

## Conclusion

We think that the self-limiting nature of our finding and others proves that certain regions have ecological, geographical and climatological features that make it difficult for the H5N1 virus to spread [1] and cause disease at least in the large scale scenario that has been worrying human and animal health authorities during the last years.

## Methods

The Basque Country avian influenza program began sample collection and processing the fall of 2005 and has since then examined more that 3500 birds by real time RT-PCR. Of these 1700 were wild birds mainly found dead but also in a lower number taken to recovery centres or trapped for ringing. Samples consist of 1 to 3 cloacal swabs soaked in virus transport media and serum whenever possible. Dead animals of anatidae and other aquatic species are submitted to post-mortem examination if not too autolytic.

The standard testing strategy, also applied to the grebe, was to cut the swab in two, freeze and store one half, and wash the other in 500 μl TE (ph:8) to recover any virus present. The whole 500 μl are submitted to RNA extraction with Qiagen RNeasy^® ^Mini Kit using 35 μl of RNAse-free water to elute RNA in the final step. The first detection step is a screening with influenza A type matrix gene RRT-PCR and if there is the slightest increase over the positive control defined-threshold, an H5 and H7 subtype specific RRT-PCR is performed with the remaining RNA [2]. Once H5 is confirmed in this PCR, a H5N1 conventional multiplex RT-PCR is run (Veredus Laboratories^©^). All reactions are set up with 5 μl of the final RNA solution. Thus, approximately one fourteenth of the total amount of the original cloacal swab is included in each RRT-PCR reaction.

## Authors' contributions

IA and II performed the field work and collected the samples of the study. VA carried out the laboratory work and MB the sequences study. RAJ and MB designed, coordinated and supervised all the study. RJU and MB wrote the manuscript. All authors read and approved the final manuscript.
